# Monthly Summary

**Published:** 1898-05

**Authors:** 


### Monthly Summary.

LIQUEFIED AIR.—The economical liquefaction of air in large quantities has been recently accomplished by Mr. Chas. E. Tripler, of New York, after several years of experimental work. Two and a half gallons of the liquid were recently sent from his laboratory to Prof. Barker, of the University of Pennsylvania, and its properties were exhibited in an extremely interesting series of experiments during a lecture delivered by Prof. Barker to his class and a company of invited guests. This was the first public exhibition of the kind of this article in the United States.

Mr. Tripler's method of liquefaction is based upon the fact that, if a gas be compressed and allowed suddenly to expand, it absorbs the heat of the surrounding medium, thereby producing intense cold. He compresses air to 2,000 pounds to the square inch, passes it through a coil and permits it to issue from a needle point orifice. There it expands and cools. This cold stream of air circulates around a second coil through which compressed air is flowing, reducing the temperature of the latter. The air issuing from this second coil has its temperature lowered to a point due to its own expansion plus the cold imparted from the first expansion. The expanded and extremely cold air from the second coil is used similarly to cool a third coil, the air in which is brought down to a temperature of 311.8° F. and below, at which it condenses and flows from the end of the coil in a liquid stream.

In the course of his lecture Prof. Barker made a number of curious experiments with the liquid, illustrating the operation of the laws governing the formation of solids, liquids and gases. When it was poured into a tumbler it boiled until it had absorbed the heat of the glass. The cold gas given off condensed the moisture in the air above the glass, which fell in the form of hoar frost. A piece of tin thrust into the liquid made it boil and the tin was rendered as brittle as glass. Copper and platinum were not so affected, and it is evident that these metals will make suitable receptacles for this new liquid. When it was boiled over a furnace the ebullition was, of course, excessive; but the moment water was poured into the boiling liquid, the former was instantly frozen. Alcohol and mercury were frozen when brought in contact with the new product. The liquefaction point of the two constituents of air is different, that of oxygen for given pressures being several degrees higher than that of nitrogen. Hence, as the temperature of the liquid rises, the nitrogen is the first to escape as a gas. The remaining liquid is proportionately rich in oxygen—a fact which is proved by the bluish tint which a standing vessel of the liquid assumes if exposed to the air. Just what the economic value of this new and extremely interesting product is time will show; but in experimental work in the laboratory it will be certain to find a ready field of usefulness.—*Scientific American.*

---

DISHONEST DENTISTRY.—When dentistry is dishonest, and that is not rare, it is more dishonest than stealing. Would it not be better for both patient and dentist if the latter were to take surreptitiously from the patient's pocket three dollars than to make a hole in a tooth and fill it for that amount? Not long since we noticed five small fillings in the morsal surface of a molar, for which, no doubt, a liberal fee was collected for each, when one crucial filling would have been better, and would have cost one half of the five fillings. Was it honest? Wasn't the extra charge for extra fillings stealing? There is hardly another profession in which the patient is so completely at the mercy of the attendant.

We quote from Dr. Burket in the *Western Dental Journal* as follows:

"The practice of crowning teeth is reaching such magnitude that it may well claim our serious attention. Scores of good teeth that might be made serviceable for years by filling are ground down and crowned, till we "lift our hands in holy horror" at the dental offices becoming dental slaughterhouses. I know personally of a case in which a dentist insisted upon crowning four teeth, only two of which had cavities larger than a pinhead, and the cavities in those two were not bad ones. To fill them would have cost but a few dollars; to crown them a number of dollars. Draw your own conclusions as to the dentist's motive. And this kind of work is going on all the time and all over the country. Shame upon the men who will sacrifice humanity for such a selfish motive! Having had the technical training that would enable them to do the right kind cf work, they are willing to injure their patients and debase the profession for the sake of gain. Measured by the standard of ethics, they are found wanting. What do we practice dentistry *for* any way? *Just* for the money that is in it, or for the good of humanity? The fact that we must earn our living through our practice does not do away with that other and greater fact that dentistry, as a profession, owes its existence to humantty's needs. When an operator's highest thought in his work is the money he can get out of it, numberless evils edge their way into his practice, almost without his notice, and he begins to degenerate."—*Am. Dent. Weekly*.

PYORRHEA ALVEOLARIS.—In discussing this subject before the Southern Dental Association, Dr. C. N. Pierce said.

"We too often make the mistake of taking the symptoms for the disease. Pus is simply an expression of pyorrhea.

"By pyorrhea alveolaris we mean 'pus in the socket." It is a conspicuous symptom and we seek to eliminate that condition.

"Five years ago, I noticed in connection with this condition of pus in the sockets other systemic conditions, and by continued observation I found that in four-fifths of the cases I also had some expressions of a gouty condition of the uric acid diathesis. I took the accumulation from the roots of the teeth to Professor Congdon, and asked him to analyze them carefully. He reported that they consisted largely of urate of soda, urate of lime and uric acid crystals. I said to the patient, I wonld suggest that you begin systemic treatment for the uric acid diathesis; place yourself on a diet as though for a case of gout. I also prescribed lithia and hot water. You cannot imagine my joy when I found the gums healing up at once with no local treatment.

"Treating the patients for gout cured the pyorrhea, but pyorrhea does not stay cured. You can arrest the progress of the disease, stop the accumulations of pus; but let your patient indulge in elaborate dinners and suppers, eat heartily of meat and drink wines, and he must suffer for it."—*Extract Dental Cosmos.*

---

CARIES VS. EROSION.—Dr. E. Noyes.—Dr. Cattell speaks of caries as necessarily being a death of the animal matter, the destruction of the vitality of the tissue bfore the integrity of the tissue was affected, and he made that as a distinction between caries and erosion. It seems to me this is not exactly a clear way of looking at it. I regard the distinction between caries and erosion as fundamental, that is, in caries you always have a softening of the surface attacked, erosion you never have a softening of the surface attacked. In caries you always have first the inorganic portion of the tooth substance attacked and dissolved out and the organic used as food by the micro-organisms. In erosion the surface is always perfectly hard.

In regard to the etiology of dental caries it seems to me that we can regard the subject as standing in a negative position.

To look back over the history of dental caries it has had several other such periods before. It was first suggested that the destruction of the tooth substance is caused by acids, then that the acids are formed at the point attacked, and the question is asked, where do the acids come from and what are they ?

In this position the knowledge on the subject stood still for years, till the development of science in regard to micro-organisms was sufficiently advanced to prove that the development of the acid was due to the vital action of micro-organisms at the point attacked.

That caries of the teeth is caused by the action of micro-organisms, it seems to me, has been proven and is established ground; it is no longer a theory but a fact. We have come again to a negative position which is perhaps stated in this way. What modifies the action of the micro-organisms so that in one month they attack the teeth, in another they do not; they exist in all cases. In the same individual, at one time they attack the teeth, at another they do not. That micro organisms destroy the tissues we know, but why in one case where we would expect them to they do not, and in another where we would expect them not to they do, we do not know. So much we know, so much we do not know.

The work of Dr. Black and Dr. Williams shows that the resistance of the teeth to caries is not caused by their chemical composition, nor is it due to the perfection of their histological structure, but that it must be due to their environment, the conditions surrounding the teeth which affect the vitality of the micro-organisms, or their action in some way. In regard to these modifications of action we know nothing as yet, that subject is to be developed. We stand in a position to ask questions, not to answer them. The questions can only be answered by work, and that work has not yet been done. *Dental Review.*

SAUCER-SHAPED CAVITIES.—By Dr. H. A. Cross.—Speaking of saucer-shaped cavities reminds me of something which I wish to present to-night for the purpose of demonstrating manipulation. You will remember that Dr. Hewett read a paper not many months ago before this society in which he recommended a method for putting in amalgam fillings, and some of you may recall the expression he used, namely, of "burnishing the amalgam into the tubuli." That attracted my attention, and in my own experience I have found so many cavities where I could not get an undercut, that if there is any method that I can adopt which will enable me to anchor a filling there without the undercut, I shall be glad to learn it. I have been practicing the method described by Dr. Hewett and as recommended by him. I am favorably impressed with it. I have here two small pieces of ivory. I took a bur, made a saucer-shaped depression, being particular not to make an undercut, and I filled those with amalgam after the plan recommended by Dr. Hewett. You will find on the side of the ivory a little depression of the same form as these fillings are placed in. If you remove the amalgam you have a test for whatever it is worth regarding manipulation, for I think the value of any filling material depends on manipulation. One dentist can put in a good gold filling, while another cannot do it to save his life. As dentist we should experiment with the different kinds of filling materials as well as with the cavities.—*Dental Review.*

---

OPENING THE BITE WITH CAP-FILLINGS, PRESERVING THE VITALITY OF THE PULP.—M. F. Finley, D. D. S., Washington, D. C.—A male aged 62 years had every tooth present in the upper jaw, and all in regular position, except the left cuspid, which closed inside. The teeth were so peculiarly worn that when the jaws were closed the upper incisors came in contact with and pressed upon the lower gums, the incisive edges of the lower incisors also impinging on the upper gums when the teeth were closed. The outer cusps of the upper molars also came in close contact with the gums of the lower jaw, forcing the food into painful contact with the gums, causing great discomfort. The molars being sound, with living pulps, it was decided to open the bite by means of cap fillings upon the second bicuspid and first and second molars on the left side lower jaw, the second bicuspid and third molar right lower heavy crowned to carry a bridge to replace the lost first and second molars, the occlusion being raised to correspond with the opened bite.

The occlusal surfaces of the teeth to be capped were ground to nearly a plane surface, and caps adjusted by means of platinum pins entering four holes in the molars and three in the bicuspids, very carefully located in the centers of the sides, avoiding the cornua. The caps and bridge were all cemented to place at one time to avoid irritation by pressure on a single tooth or on one side of the jaw.—*Ohio Dental Journal.*

---

A MYSTERIOUS STAIN.—Dr. J. A. Stewart, Longview, Texas.—I have a peculiar case of discoloration on the face of the upper incisors of a patient. It will not polish off, and I have tried chlorid of lime and acetid acid, but it has no effect. The teeth have no cavities in them, and are alive, good teeth. I am at a loss to know what to do. The discoloration is of a dark brown, and has been on them for years. The young man is of about nineteen or twenty years. He says he does not know what caused it unless it was concentrated lye, which he took into his mouth by mistake when about nine years of age, and which came very near killing him from what he swallowed. Do you know any thing by which I could bleach them without injury to either the pulp or enamel.—*Dental Brief.*

---

To painlessly cure a corn first keep it wet through the night, and then, after picking off all the soft part, apply a cotton dressing saturated with vaseline, covering this with a patch of rubber adhesive plaster. Repeat this daily, each time removing what can be easily detached. The corn or wart will gradually disappear.

THE USE OF NON COHESIVE GOLD.—Dr, C. N. Johnson.—As to the use of non-cohesive gold along the cervical margin, as advocated by many members of the profession, it is used in such a place, not so much because we cannot get adaptation with cohesive gold, but because in placing a layer of non cohesive gold and condensing the cohesive gold upon it we have a cushion for the cohesive gold to be condensed on, and can mallet over that part of the filling with less danger of injuring the enamel margin than we could if we started with cohesive gold and began our condensation in the early part of the operation. That small piece of non cohesive gold serves as a cushion to protect the margin from injury from the malleting force. We do not want to use much non-cohesive gold in building up proximal fillings in molars and bicuspids. The only non-cohesive gold I would use would be the first layer. From that point up I would use cohesive gold on account of its greater density. But I do not want at this time to enter into a discussion of the relative merits of non cohesive and cohesive gold.—*Dental Review.*

---

IMMEDIATE INVESTING MATERIAL.—A nice and convenient way to make an investment for soldering, is to take a small quantity of asbestos fiber, place it in a dry rubber bowl and incorporate with the fingers as much of Teague's impression material as the asbestos will take up. Moisten the compound as you use it, and shape the investment with the fingers as desired, around the piece to be soldered. The investment becomes hard immediately on being heated, and does not change its shape Plaster will not do as a substitute for Teague's material, as it changes its shape on heating, even when mixed with asbestos.

In using to invest pieces of work held together by wax, the wax should always be removed by warming the investment and picking it out with the point of a suitable instrument. Hot water poured on the investment softens in on account of the asbestos fiber absorbing the water. Porcelain faces can be invested with this mixture and dried out immediately with the flame of a blowpipe without the danger of cracking them.—*H. R. J. in Amer Dental Weekly.*

TAKING DIFFICULT IMPRESSIONS.—Several years since Dr. E. Angle gave to the profession the following method for taking difficult impressions:

Impressions of the mouth for a full or partial denture should always be taken in plaster. Where difficulties arise, as they often do in partial cases, oil the impression cup before pouring the plaster, to facilitate the removal of the former from the latter, then divide the outer portion of the impression into three pieces, when the whole can be easily removed and replaced in the cup. After obtaining a good impression of an upper, which must include a part of the soft palate and condyles of the jaw, the portion of the plaster indicating the location of the hard palate—unless of a soft, spongy membrane or tissue over the hard palate—should be trimmed to relieve pressure at that point. Knock the impression out of the cup, trim off all surplus plaster before pouring the plaster for the model.

In case the plaster impression, after its proper preparation, in a sheet of lead two and one-half inches wide and about 24 gauge, the object of this is obvious. The resulting model should be scraped to the depth of a line on that part representing the soft palate, then with proper articulation of the teeth, satisfaction for the patient and dentist usually ensues.

---

MIXING AMALGAM.—Dr. I. N. Crouse.—It is very essential to have the proper proportion of mercury and amalgam when you commence mixing. Two ways have been suggested for doing this. One is to weigh out the mercury and amalgam and put them in capsules, so many grains of amalgam and so much mercury to go with it. This method is objectionable because too complicated. The other plan is to have a scale with which the assistant can soon learn to weigh out a sufficient amount for the size of cavity. You soon are able to gauge accurately the number of grains of amalgam that are required to fill the cavity. Be sure to have enough mercury, so as to mix the amalgam to its proper consistency. All amalgam should be mixed softer than many operators think. Some have their amalgam too dry and they do not stand the stress. An amalgam that will stand a stress of from 350 to 450 pounds, if mixed dry will break at 80 or 100 pounds. The mixing of amalgam is so important that it is much better to have it too wet than too dry. There is also such a thing as wringing it out drier than it should be. If it is too dry it begins, to powder and harden before it is in place. I have adopted the method of having it moist, and then take some dry amalgam and incorporate with it.—*Review.*

---

DR. EVANS.—It is but natural that many errors should be made in the various biographical sketches published of Dr. Thomas W. Evans. His fortune has been absurdly exaggerated, and the final disposition of it has been confidently stated by those who had nothing but baseless rumor to guide them. Perhaps the most common mistake is in saying that he was even the professional adviser of Queen Victoria. For many years her dentist has been Sir Edwin Saunders, and she is not the kind of woman to call in a foreigner under any circumstances, no matter what might be his accomplishments. But Dr. Evans was personally well known to her and was employed by members of her family. He was never "Court Dentist" in any country outside France, though he was often called in consultation, and to perform special service in all the capitols of Europe.

---

IN nearly all exposed pulps, and sometimes when they are entirely exposed, a paste of iodoform and creosote carefully laid on over the exposure is a good covering to precede oxi-phosphate. A little of the powder of the cement mixed with equal parts of oil of cloves and creosote is alzo good.

---

"O, I can never go to that man again. He has the gravity and coldness of a hangman." So said a lady to whom I had recommended a really skilled dentist. It is a pity some men do not know how to receive their patients, and entertain them affably after they are received.

